# Up-regulation of long noncoding RNA MBNL1-AS1 suppresses breast cancer progression by modulating miR-423-5p/CREBZF axis

**DOI:** 10.1080/21655979.2022.2026728

**Published:** 2022-01-30

**Authors:** Jun Fang, Gaohui Jiang, Weiguo Mao, Lili Huang, Chen Huang, Shanshan Wang, Huimin Xue, Jing Ke, Qichao Ni

**Affiliations:** a Department of General Surgery, Affiliated Hospital of Nantong University, Nantong, Jiangsu Province, China; b Department of Critical Care Medicine, Affiliated Hospital of Nantong University, Nantong, Jiangsu Province, China

**Keywords:** lncRNA, MBNL1-AS1, breast cancer, miR-423-5p, PI3K/AKT

## Abstract

Breast cancer is the leading cause of cancer-related death among females, which is required to be solved urgently. Recent studies have found significant changes in a large number of genes and their transcriptional levels during breast cancer development, which are often closely related to the abnormal expression of long noncoding RNAs (lncRNAs). Herein, our study found that MBNL1-AS1 was down-regulated both in breast cancer tissues and cell lines, and it functioned as a tumor suppressor to inhibit cancer cell proliferation, migration, and invasion. MiR-423-5p was found to be a target of MBNL1-AS1 with an inverse relationship: an increase in miR-423-5p could counteract the inhibitory effect induced by MBNL1-AS1 on cancer cell promotion. Further, CREBZF was negatively regulated by miR-423-5p. Accordingly, CREBZF knockdown could impair the hindrance of cancer cell growth mediated by low miR-423-5p expression. Also, MBNL1-AS1 influenced the PI3K/AKT pathway, which was associated with cell proliferation and apoptosis, by regulating CREBZF. As a result, our work illustrated the tumor suppressor role of MBNL1-AS1 in breast cancer via upregulating miR-423-5p-targeted CREBZF. Thereby, the evidence indicates the complete understanding of the role of MBNL1-AS1/miR-423-5p/CREBZF axis in the regulation of breast cancer development, which could be used as a biomarker for predicating survival among breast cancer patients.

## 1. Introduction

Breast cancer is the most commonly diagnosed cancer worldwide, and is also the leading cause of cancer-related death among females. Among American women, breast cancer is the second leading cause of cancer-related death after lung cancer[1]. Current therapeutic methods for breast cancer contain surgical resection, chemotherapy and radiotherapy, endocrine therapy, targeted therapy, immunotherapy, and combination therapy. It still has a high mortality and recurrence rate. Mutations of genes and abnormal expressions of proteins accompany the development of breast cancer; therefore, the exploration of tumor biomarkers is essential for cancer detection, diagnosis, and therapy. Meanwhile, a complete understanding of the molecular mechanisms underlying carcinogenesis and cancer progression in breast cancer is needed.

Long noncoding RNA (lncRNA) is a type of functional RNA containing at least 200 nucleotides, which was first discovered in mouse DNA transcription products by Okazaki et al [2]. LncRNA has spatiotemporal specificity: the expression of lncRNA changes at different growth stages of the same tissue or organ [3,4]. LncRNAs play crucial roles in tumor development. Therefore, it is very important to identify cancer-related lncRNAs and explore their biological function and molecular mechanism [5,6]. LncRNA muscle blind-like 1 antisense RNA 1 (MBNL1-AS1) is one of the hottest topics in cancer research related to aberrant lncRNA expression. Previous research has pointed out that MBNL1-AS1 is down-regulated in colorectal cancer cells and that it functions as a cancer suppressor gene by targeting miRNAs such as miR-1307 and miR-412-3p [7,8]. In bladder cancer cells, lncRNA MBNL1-AS1 inhibits cancer cell proliferation and migration both *in vivo* and *in vitro* by mediating miR-362-5p/QKI axis [9]. However, the role of lncRNA MBNL1-AS1 has not been studied thoroughly in breast cancer.

Competitive endogenous RNA (ceRNA) hypothesis was first proposed by Salmena et al., who suggested that lncRNA could adsorb miRNA through miRNA response elements (MRE), thereby inhibiting the effect of miRNA and indirectly regulating the expression of protein coding genes [10,11]. Little investigation has been conducted on the down-stream modulatory factor of MBNL1-AS1 in breast cancer. MiR-423-5p is now acknowledged to be a key factor that promotes the progression of various cancer types. Our study exhibited that miR-423-5p may be a crucial down-stream modulatory factor of lncRNA MBNL1-AS1. This finding further promotes the understanding of MBNL1-AS1 in breast cancer.

CREBZF, a member of the mammalian ATF/CREB family, is a transcription factor that participates in the progression of multiple live movements. It has been reported that CREBZF also partakes in the regulation of p53 and thereby modulates cell death [12]. However, the specific function of CREBZF in carcinomas, especially in breast cancer, has not been investigated. Herein, this study aimed at disclosing the specific function and the underlying mechanism of lncRNA MBNL1/miR-423-5p/CREBZF axis in promoting breast cancer progression, so as to provide a potential novel biomarker for predicating the survival of breast cancer patients.

## 2. Materials and methods

### 2.1. Patient tissues

Breast cancer tissues and normal tissues of patients were collected from Affiliated Hospital of Nantong University between January 2015 and December 2015. The extracted sample tissues were washed with sterile saline, immediately rapidly frozen in liquid nitrogen, and then stored at −80°C for further analysis. All experiments were approved by the ethics committee of Affiliated Hospital of Nantong University, and all patients signed the informed consent form.

### 2.2. Cell culture and transfection

MCF-10A, BT474, MDA-MB-231, MDA-MB-453, ZR-75-30, and MCF-7 cells were cultured in DMEM containing 10% FBS, at 37°C, with 5% CO_2._ When the degree of cell fusion reached 80%, 0.25% trypsin was used for digestion and passage. pcDNA 3.1 plasmids with full length MBNL1-AS1 (MBNL1-AS1 ov) and related negative controls (NCs) were used to upregulate MBNL1-AS1 in the MCF-7 cell line. Small interfering RNAs against MBNL1-AS1 (si-MBNL1-AS1), as well as small interfering RNA negative controls (si-NCs), were cloned into PGK plasmids and used to knockdown the expression of MBNL1-AS1 in the BT474 cells. The miR-423-5p inhibitor and mimics were also used to regulate the expression of miR-423-5p. si-CREBZF was used to downregulate CREBZF levels in MCF-7 cells. Cells in the logarithmic growth phase were inoculated with the density of 2 × 10^5^ in a 6-well plate for culture, and transfected when the cell density reached approximately 80%. The cells were then incubated in an incubator at 37°C, 5% CO_2_, for 4 h. The original medium was replaced with DMEM for culture. The procedure was followed based on the assay instructions of Lipofectamine^TM^ 2000.

### 2.3. Quantitative reverse transcriptase polymerase chain reaction (qRT-PCR)

The expression levels of MBNL1-AS1, miR-423-5p, and CREBZF mRNA were detected via qRT-PCR. Total RNA was extracted from fresh clinical samples and breast cancer cells using the TRizol reagent following the guidelines. The RNA was reverse-transcribed into cDNA. The expressions of MBNL1-AS1 and CREBZF were detected using the ABI 7900HT RealTime PCR System by SYBR Green assays, and GAPDH was used as an internal control. The expression of miR-423-5p was measured by TaqMan MicroRNA Assays, and U6 was treated as an internal control. The relative expression was calculated by 2^−ΔCt^ and 2^−ΔΔCt^ method.

### 2.4. Western blotting

The total protein of clinical samples and breast cancer cells was extracted using the RIPA lysis buffer, and quantitatively detected using BCA protein detection kit. Further, 20 μg protein sample was added to the well, and the protein was separated by 12% polyacrylamide gel electrophoresis, and transferred to a PVDF membrane. Next, 5% skimmed milk powder was added to seal at room temperature for 1 h, and the primary antibodies (CREBZF, p-PI3K, t-PI3K, p-AKT, t-AKT, Bcl-2, Vimentin, and CyclinD1) and internal control mouse anti-GAPDH (1:3000) were added, followed by incubation overnight at 4°C, washing 3 times with PBS, for 10 minutes each time, and the addition of the secondary antibody (1:5000). This was followed by incubation for 2 h at room temperature, and washing 3 times with PBS, for 10 minutes each time. The enhanced chemical luminescent (ECL) solution gel was added to the imaging system for detection; the image analysis software was used to calculate the gray value of each band.

### 2.5. CCK-8 assay

The transfected cells in each group were inoculated into a 96-well plate at a density of 2 × 10^3^ cells/well in 100 μL medium, followed by incubation at 37°C, 5% CO_2_. The CCK-8 kit was then used to detect the cell proliferation ability. Herein, 10 μL CCK-8 solution was added to each well at 0, 24, 48, 72, and 96 h, then the plate was placed in a cell incubator to continue incubation for 2 h, and the absorbance value of each well was detected at 450 nm on a microplate reader [13].

### 2.6. Colony formation assay

Each group of cells was pipetted into a single-cell suspension state, 1 × 10^3^ cells were inoculated into a plate, shaken gently to make cells evenly disperse, and incubated at 37°C, 5% CO_2_, for 2 weeks; visible cell colonies were observed. After washing with PBS, 4% paraformaldehyde was added to fix the colonies, followed by staining with 0.5% crystal violet for 15 min, rinsing under running water, and drying under natural conditions. The plates were observed under a light microscope. The 50 collective cells were seen as 1 cell colony, and the relative number of cell colonies was calculated.

### 2.7. Flow cytometry

The logarithmic growth phase breast cancer cells were collected from each group, washed with pre-cooled PBS, digested using trypsin, centrifuged at 10,000 r/min for 5 min at 4°C, and washed with PBS; 1 mL binding buffer was then added, followed by centrifugation under the same conditions. The supernatant was discarded, and 200 μL binding buffer and 5 μL annexin V-fluorescein isothiocyanate and propidium iodide were added. This was followed by incubation at room temperature for 20 min in the dark, and addition of 400 μL binding buffer. The apoptosis rate was detected by flow cytometry.

### 2.8. Transwell assay

Cell invasion assay: 50 μL diluted Matrigel glue was added to the upper transwell chamber and placed in an incubator at 37°C for 30 min. After transfected cells were adjusted for 2 × 10^4^/mL, 100 µL of cell suspension was added to each well of the upper chamber, and 500 µL DMEM containing 10% FBS was added to the lower chamber to culture until the cells adhere to the wall. The serum-free culture medium was changed and incubated for 12 h at 37°C, 5% CO_2_. The cells were then moved, the culture medium was discarded; the cells in the upper chamber were gently wiped, fixed with 4% paraformaldehyde for 10 min, stained with 0.1% crystal violet for 10 min, and observed under optical microscope; the number of invaded cells was counted. For the migration assay, the step of enfolding the upper chamber with Matrigel was skipped, and the rest of the procedures followed the same guideline.

### 2.9. Wound healing assay

Transfected cells were seeded in a 6-well plate at 4 × 10^5^ cell/well and cultured for 24 h. A pipette tip was used to make a mark along the straight edge perpendicular to the middle of the cell culture well. The cells were washed with PBS 3 times. The cells under the scratch were then added to serum-free DMEM and placed in a cell incubator at 37°C, 5% CO_2_, to continue culturing. The healing of the scratch was then observed under an optical microscope at 0 and 48 h and pictures were taken; an image analysis software was used for the analysis [14].

### 2.10. Dual-luciferase assay

The binding sites of MBNL1-AS1 and CREBZF with miR-423-5p predicted by bioinformatics tool were amplified by PCR. The amplified products were inserted into pGL3 plasmid to construct wild-type (wt) plasmids of MBNL1-AS1, and CREBZF, MBNL1-AS1, and CREBZF mutant (mut) plasmids were constructed by site-directed mutagenesis of the combined fragments by gene mutation techniques. MCF-7 cells were inoculated in the logarithmic growth phase on a 12-well cell plate at 1 × 10^5^ cells/well, and Lipofectamine 2000 to combine the constructed 100 ng dual luciferase recombinant plasmid with 100 nM miR-423-5p mimics or miR-NC. After transfection, the cells were cultured in a cell incubator at 37°C, 5% CO_2_, for 48 h. The luciferase activity in each group of cells was detected according to the steps provided in the dual luciferase reporter kit.

### 2.11. Animal experiment

BALB/ cA-Nu nude mice, aged 4 to 5 weeks and weighing between 16 g and 18 g, were purchased. At room temperature (between 25°C and 27°C), with constant humidity (45% to 50%), and fresh sterilized filtered air, dust removed and sterilized without special pathogenic bacteria were fed in a feeding room for 7 days for an adaptive period, and then subcutaneous tumor cells were inoculated. The concentration of the cell suspension was allowed to reach 1 × 10^7^ /mL with PBS. Following this, 100 μL of the cell suspension was subcutaneously injected into the back and upper part of the nude mice. The living status and tumor formation in mice were observed every 4 days regularly. Further, 20 days after inoculation, the experimental animals were killed, and the subcutaneous tumor tissue was completely removed. The tumor images and weights were recorded; the tumor average volume was calculated as 1/2 × length (mm) × width (mm)^2^

### 2.12. Statistical analysis

The SPSS 20.0 and GraphPad 8 statistical software was used for data analysis. Each experiment was repeated 3 times independently; the measurement data is represented as mean ± standard deviation. The t-test was used for comparison between two groups, and one-way analysis of variance was applied for comparison between multiple groups. Kaplan–Meier and log-rank analysis were used for survival analysis. P < 0.05 indicated statistically significant difference.

## 3. Results

### 3.1. Decreased lncRNA MBNL1-AS1 level was detected both in breast cancer tissues and cell lines

**Figure 1.**
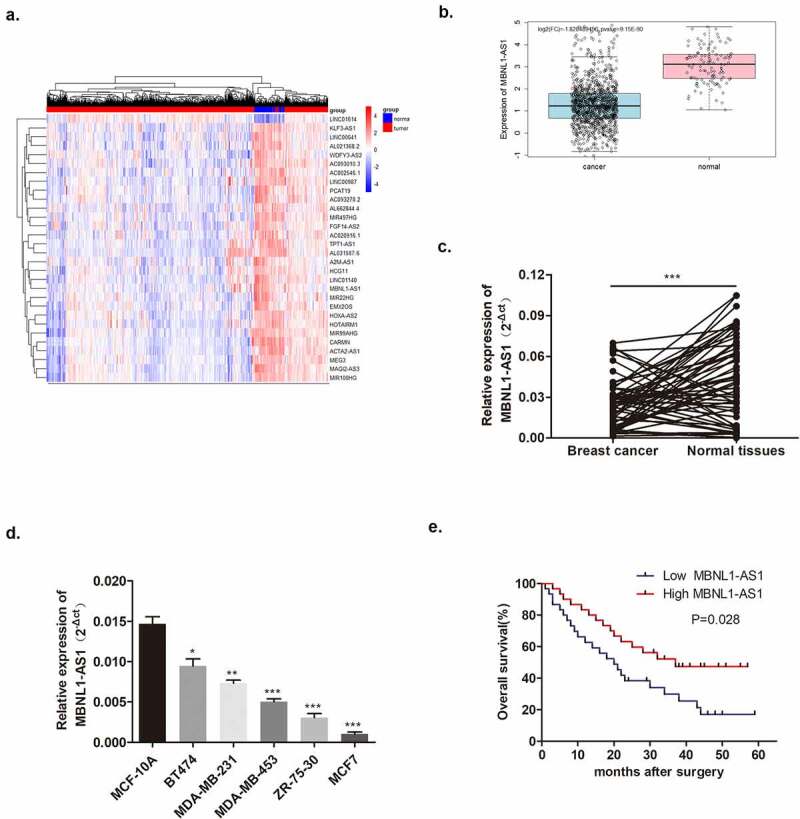
LncRNA MBNL1-AS1 was downregulated in breast cancer tissues and cell lines. (a) The heatmap of differentiated lncRNAs in breast cancer screened via TCGA database. (b) The bioinformatics prediction from TCGA database showed that MBNL1-AS1 showed lower expression levels in breast cancer tissues compared with normal tissues. **C**. Boxplot exhibits that MBNL1-AS1 in 60 cancer tissue samples was decreased compared with normal tissues. (d) MBNL1-AS1 level in five breast cancer cell lines (BT474, MDA-MB-231, MDA-MB-453, ZR-75-30, and MCF-7) was poorer than that in normal MCF-10A cells (MCF-7 showed the lowest level, and BT474 showed relatively high MBNL1-AS1 levels) by qRT-PCR; (e) Five-year survival of patients with high expression of MBNL1-AS1 was higher compared with patients with low expression of MBNL1-AS1.

As mentioned before, abnormal lncRNA expression was common in cancers. The heatmap showed aberrant expressions of lncRNAs in breast cancer from TCGA database screening and significantly decreased MBNL1-AS1 levels were detected in breast cancer tissues compared with normal tissues (Figure 1a,b). Based on the data from 60 pairs of breast cancer patient tissues at our hospital, we confirmed that lncRNA MBNL1-AS1 was poorly expressed in cancer tissues than in normal tissues (Figure 1c). Simultaneously, lower lncRNA MBNL1-AS1 level was also observed in five breast cancer cell lines (BT474, MDA-MB-231, MDA-MB-453, ZR-75-30, and MCF-7) compared with normal breast epithelium cell line MCF-10A (Figure 1d). Furthermore, on evaluating the relevance of MBNL1-AS1 level with overall patient survival, we observed that the five-year survival of the patients with high expression of lncRNA MBNL1-AS1 was significantly better than that of the patients with low expression of lncRNA MBNL1-AS1 among the 60 patients (Figure 1e). The primary evidence indicated that lower MBNL1-AS1 level was related to breast cancer occurrence and survival rate. Furthermore, we studied the relationship between MBNL1-AS1 expression and the clinicopathological features of breast cancer patients. As shown in Table 1, MBNL1-AS1 expression was significantly correlated with lymph node metastasis (P = 0.020) and pathological type (P = 0.011). However, it was not related to age, tumor size, histological grade, estrogen receptor, progesterone receptor, HER-2, or Ki67.

**Table 1.** Correlation of MBNL1-AS1 expression with clinicopathological characteristics in BC patients

characteristic	case	MBNL1-AS1 expression		*P* value
		low	high	
All case	60	30	30	
Age (years)				0.787
<60	39	20	19	
≥60	21	10	11	
Tumor size (cm)				0.166
<2 cm	10	3	7	
≥2 cm	50	27	23	
Lymph node metastasis				***0.020***
Negative	16	4	12	
Positive	44	26	18	
Pathological type				***0.011***
Ductal carcinoma in situ	9	1	8	
Invasive ductal carcinoma	51	29	22	
Estrogen receptor				0.222
Absent	14	9	5	
Present	46	21	25	
Progesterone receptor				0.754
Absent	13	6	7	
Present	47	24	23	
HER-2				0.243
Negative	16	10	6	
Positive	44	20	24	
Ki67				0.317
<14%	11	7	4	
>14%	49	23	26

### 3.2. Suppressive effects of MBNL1-AS1 on proliferation, migration, and invasion of breast cancer in vitro

**Figure 2.**
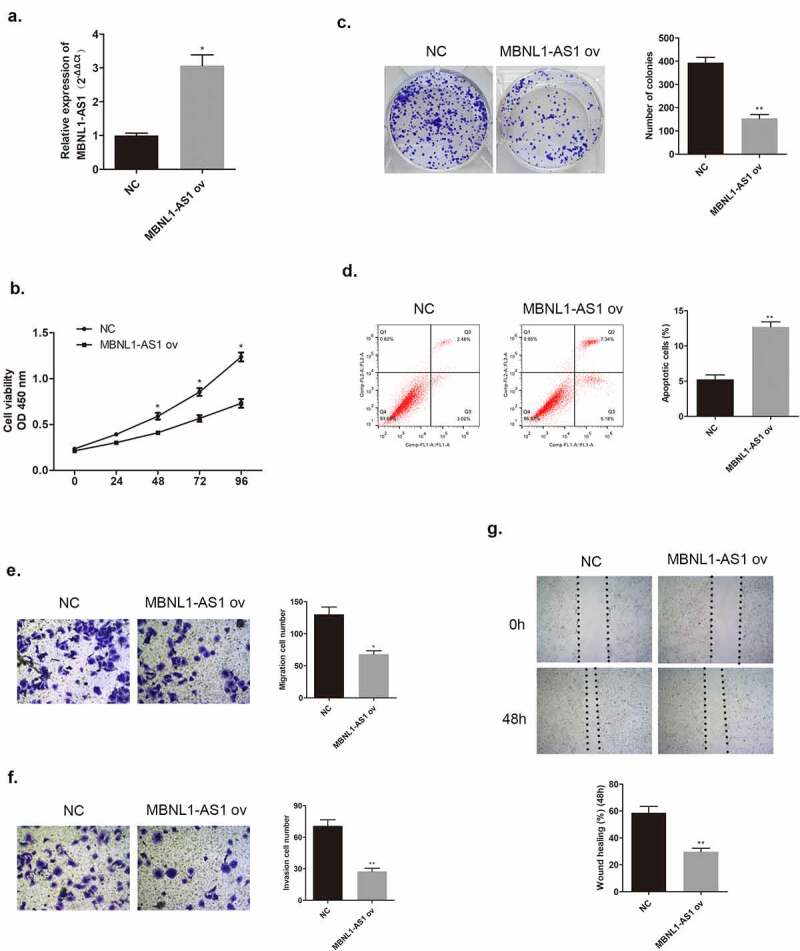
Increased MBNL1-AS1 expression could inhibit MCF-7 cell proliferation, migration, and invasion abilities. (a) Transfection efficiency by NC and MBNL1-AS1 ov was determined by qRT-PCR. (b) Cell viability in the MBNL1-AS1 ov group was significantly decreased as observed in CCK-8 assay. (c) The colony formation ability in the MBNL1-AS1 ov group was much lower than that in the NC group. (d) Apoptosis by flow cytometry showed that the overexpression of MBNL1-AS1 could notably promote the apoptosis rate in breast cancer. Cell migration (e) and invasion (f) ability in the MBNL1-AS1 ov group were conspicuously decreased compared with those in the NC group. (g) Wound healing assay further verified the attenuated migration in the MBNL1-AS1 ov group. NC, negative control, with MCF-7 cells transfected with blank plasmid; MBNL1-AS1 ov, MBNL1-AS1 overexpression, with MCF-7 cells transfected with MBNL1-AS1 overexpression plasmid. **p < 0.01, *p < 0.05.

**Figure 3.**
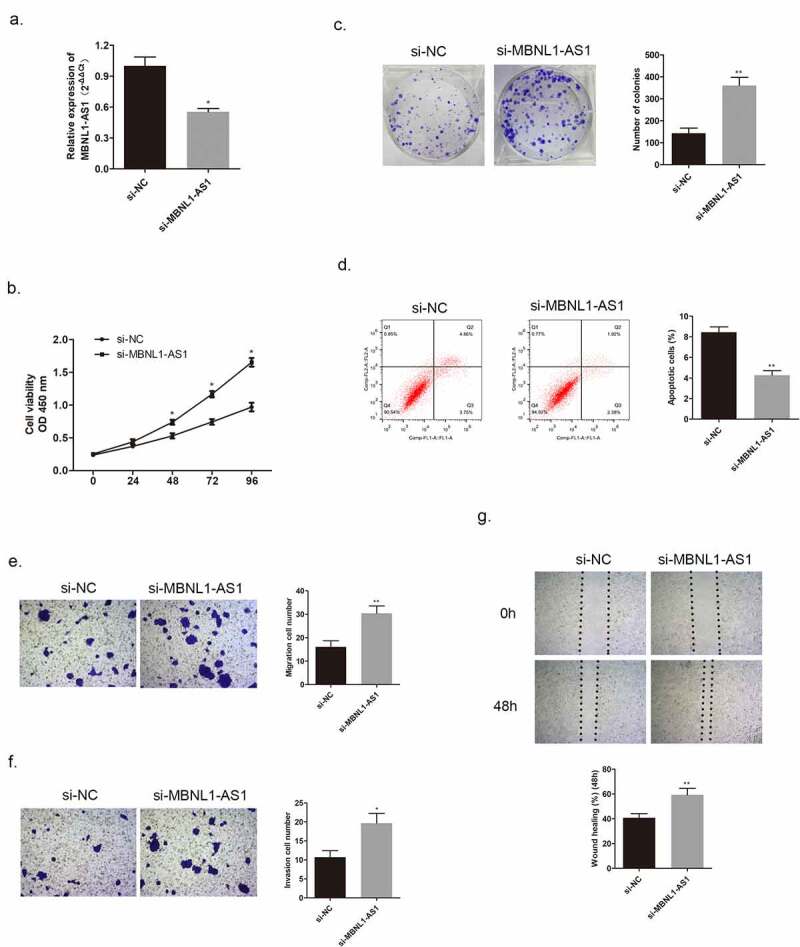
MBNL1-AS1 knockdown could promote BT474 cell proliferation, invasion, and migration abilities. (a) MBNL1-AS1 knockdown system was constructed in BT474 cell line; qRT-PCR detected MBNL1-AS1 expression after transfection. (b) CCK-8 assay was applied to evaluate proliferation. (c) Colony formation experiment was performed to determine the ability of clone formation. (d) Apoptosis determined by flow cytometry. **(E & F)** Transwell migration and invasion assays were conducted and results showed that MBNL1-AS1silencing would elevate both migration and invasion. (g) Wound healing assay further indicated that MBNL1-AS1 knockdown potentiated cell migration. si-NC, negative control of siRNA, with BT474 cells transfected with negative control of siRNA; si-MBNL1-AS1, siRNA of MBNL1-AS1, with BT474 cells transfected with siRNA of MBNL1-AS1. **p < 0.01, *p < 0.05.

In the section that follows, the specific effect of MBNL1-AS1 on breast cancer cells will be discussed. Among the five cancer cell lines, the lowest MBNL1-AS1 level was detected in MCF-7, whereas the highest MBNL1-AS1 level was detected in BT474 (Figure 1d). QRT-PCR was applied to examine MBNL1-AS1 levels in cells. Here, we evaluated MBNL1-AS1 overexpression system in MCF-7 with MBNL1-AS1 ov transfection (Figure 2a) and MBNL1-AS1 knockdown system in BT474 cell line with si-MBNL1-AS1 transfection (Figure 3a). The proliferation ability was determined via CCK-8 assay and colony formation assay. The results showed that cancer cell growth was remarkably inhibited by MBNL1-AS1 overexpression (Figure 2b,c), whereas the cell proliferative ability was promoted on MBNL1-AS1 knockdown (Figure 3b,c). Flow cytometry monitored cell apoptosis. Figure 2d shows that accelerated cancer cell apoptosis was brought about by high MBNL1-AS1 expression. However, lower MBNL1-AS1 level led to weak cancer cell apoptosis (Figure 3d). Transwell assay and wound healing assay evaluated cell migration and invasion capacities. In the MBNL1-AS1 overexpression group, the ability of cancer cell migration was attenuated (Figure 2e-g), whereas on silencing MBNL1-AS1, the enhanced abilities of cancer cell migration and invasion were observed (Figure 3e-g). The abovementioned findings firmly supported that up-regulated MBNL1-AS1 facilitated the inhibition of breast cancer promotion and migration *in vitro*, and implied that MBNL1-AS1 exerted its function as a cancer suppressor in breast cancer.

### 3.3. *MBNL1-AS1 hindered tumor growth* in vivo

**Figure 4.**
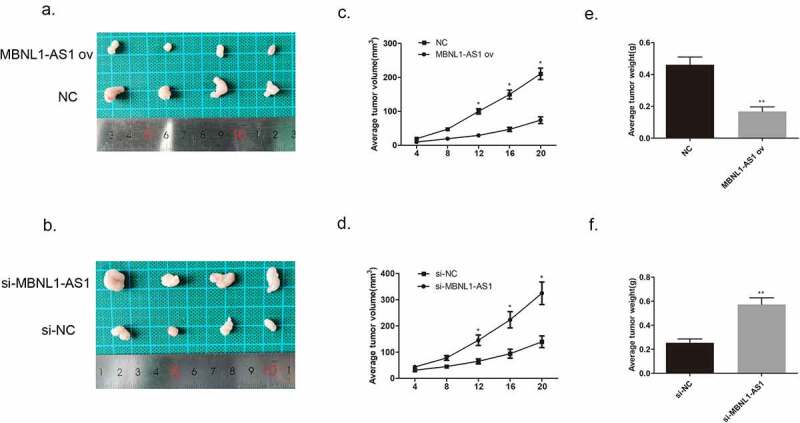
MBNL1-AS1 suppressed tumor formation and growth *in vivo*. **(a&b)** Tumor images of MBNL1-AS1 overexpression and MBNL1-AS1 knockdown groups. **(c&d)** Average tumor volume in MBNL1-AS1 ov group escalated slowly, whereas that in the MBNL1-AS1 silencing group showed a higher growth rate when compared with the NC group. **(e&f)** The weight of tumors in nude mice; MBNL1-AS1 overexpression could inhibit tumor growth, whereas MBNL1-AS1 knockdown facilitated tumor development. NC, negative control, with MCF-7 cells transfected with blank plasmid; MBNL1-AS1 ov, MBNL1-AS1 overexpression, with MCF-7 cells transfected with MBNL1-AS1 overexpression plasmid. Si-NC, negative control of siRNA, with BT474 cells transfected with negative control of siRNA; si-MBNL1-AS1, siRNA of MBNL1-AS1, with BT474 cells transfected with siRNA of MBNL1-AS1. **p < 0.01, *p < 0.05.

We injected MCF-7 cells transfected with MBNL1-AS1 ov plasmid or NC plasmid and injected BT474 cells transfected with si- MBNL1-AS1 plasmid or NC plasmid into nude mice to establish the corresponding animal models. The images of tumor xenograft are presented in Figure 4a,b. We recorded the tumor volume every 4 days. After MBNL1-AS1 ov transfection, the tumor volume and weight were markedly inhibited compared with the control NC group values (Figure 4c,e). In contrast, when MBNL1-AS1 was knocked down, the tumor growth was faster than that in the control group, which was consistent with the trend observed *in vitro* (Figure 4d,f), suggesting that MBNL1-AS1 could hinder breast cancer growth *in vivo*.

### 3.4. MBNL1-AS1 was one of targets of miR-423-5p with negative modulation

**Figure 5.**
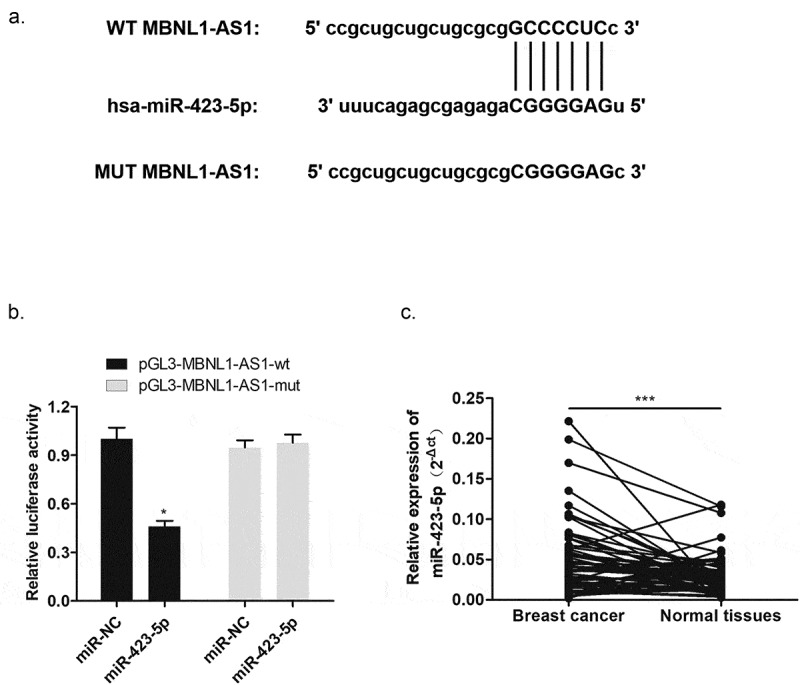
MBNL1-AS1 targeted miR-423-5p with negative regulation. (a) Bioinformatics prediction presented the binding sites between MBNL1-AS1 and miR-423-5p. (b) Dual luciferase reporter assay verified a direct interaction between MBNL1-AS1 and miR-423-5p. (c) The expression of miR-423-5p in 60 breast cancer patient tissue samples was higher than that in normal tissue samples. ***p < 0.001, **p < 0.01, *p < 0.05.

To explore the molecular mechanism of MBNL1-AS1 function, we applied bioinformatics analysis to determine the possible target of MBNL1-AS1. It has been acknowledged that lncRNA could sponge miRNA as ceRNA [15]. The intersection of prediction targets using Starbase databases showed the possible binding sites between MBNL1-AS1 and miR-423-5p (Figure 5a). Dual luciferase assay demonstrated that miR-423-5p mimics significantly reduced the activity of the luciferase reporter gene fused to the MBNL1-AS1-wt, but the change was not significant after transfection with MBNL1-AS1-mut (Figure 5b). Moreover, qRT-PCR was used to test miR-423-5p expression level in breast cancer tissues. As presented in Figure 5c, higher miR-423-5p level was observed in 60 pairs of breast cancer tissues compared to normal tissues. In brief, these results elucidated that MBNL1-AS1 directly targeted miR-423-5p via negative regulation, which may point that miR-423-5p could be a positive biomarker for breast cancer.

### 3.5. MiR-423-5p could reverse the inhibitory effect brought by MBNL1-AS1 on cancer cell development

**Figure 6.**
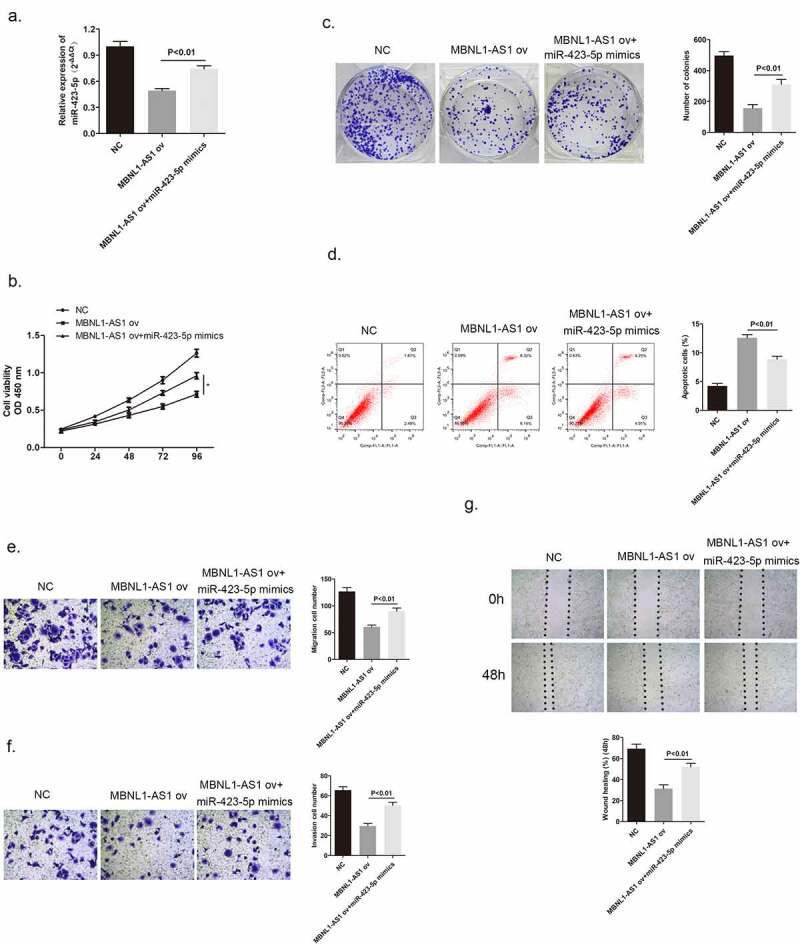
MiR-423-5p reversed the inhibitory effect of MBNL1-AS1 on MCF-7 cell promotion. (a) MiR-423-5p expression in the three groups after transfection detected by qRT-PCR (b) Cell viability evaluated by the CCK-8 assay. (c) Colony formation experiment identified the ability of clone formation. (d) Apoptosis ability assessed by flow cytometry. The abilities of migration (e) and invasion (f) were determined by transwell assay. (g) Wound healing assay was performed to describe migration capacity. NC, negative control, with MCF-7 cells transfected with blank plasmid; MBNL1-AS1 ov, MBNL1-AS1 overexpression, with MCF-7 cells transfected with MBNL1-AS1 overexpression plasmid; MBNL1-AS1 ov + miR-422-5p mimics, with MCF-7 cells transfected with MBNL1-AS1 overexpression plasmid and miR-422-5p mimics.

After confirming that MBNL1-AS1 has a direct interaction with miR-423-5p, we further evaluated the biological function of miR-423-5p in MCF-7 cells by a series of rescue experiments and determined the possible relationship between miR-423-5p and MBNL1-AS1. Figure 6a shows that miR-423-5p level was higher on co-administration of miR-423-5p mimics to MCF-7 cells with MBNL1-AS1 ov transfection compared with the administration of MBNL1-AS1 ov alone. CCK-8 colony formation assay aimed to describe the cell proliferation ability. The results revealed that MBNL1-AS1 overexpression could inhibit MCF-7 cell growth compared with the control group, which was consistent with previous findings. However, after adding miR-423-5p mimics to MBNL1-AS1 ov group, the cancer cell proliferation rate was faster than that observed in the MBNL1-AS1 ov group; this indicated that miR-423-5p reversed the inhibitory effect brought about by MBNL1-AS1 on cancer cells (Figure 6b,c). Accordingly, the cell apoptosis ability was impaired by increasing the miR-423-5p level in this group compared with that in the MBNL1-AS1 ov group via flow cytometry experiment (Figure 6d). Moreover, we conducted transwell assay and wound healing assay to evaluate the cell migration ability. The continual growth of cell migration and invasion in miR-423-5p mimics + MBNL1-AS1 ov group when compared with that in the MBNL1-AS1 ov group was clearly observed in Figure 6e–g. Collectively, the results listed above confirm that miR-423-5p could reverse biological function of MBNL1-AS1 on breast cancer cells, acting as a positive regulator in breast cancer cell development.

### 3.6. CREBZF was a negative regulator of miR-423-5p

**Figure 7.**
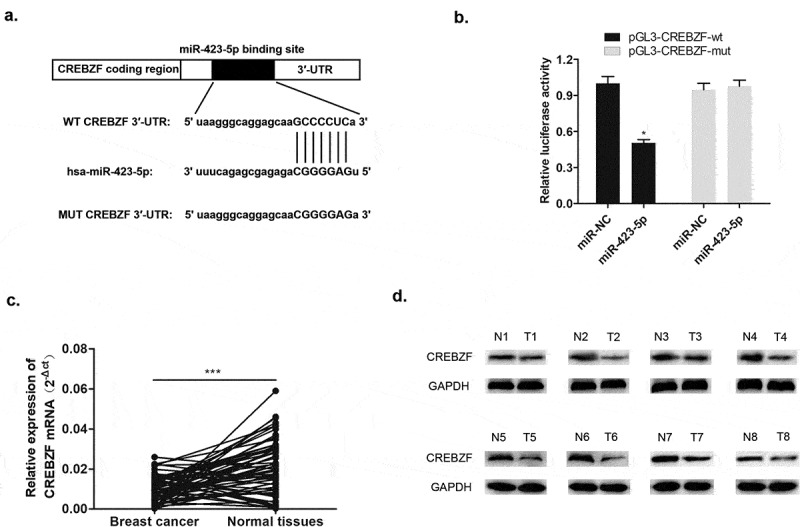
MiR-423-5p targeted CREBZF with a negative relationship. (a) Complementary sequence of miR-423-5p and CREBZF 3′UTR. (b) Dual luciferase reported assay experiment. (c) CREBZF mRNA levels in 60 tissue samples were lower than those in normal tissue samples via qRT-PCR. (d) CREBZF protein in eight pairs of selected tissue samples was poorly expressed compared with that in adjacent tissues by Western blotting.

The basic leucine zipper (bZIP) transcription factor of CREBZF is also called ZF [16]. Previous studies have shown that CREBZF plays a regulatory role in cell proliferation and apoptosis; interacts with p53, XBP1, HERP, and GRP78 to participate in the unfolded protein reaction; and regulates endoplasmic reticular stress [12,17]. Herein, through bioinformatics analysis databases including Starbase, DIANA, and TargetScan, the 3′-UTR complementary sequence between miR-423-5p and CREBZF was identified, indicating that CREBZF is the downstream target of miR-423-5p (Figure 7a). Specifically, the following dual luciferase assay demonstrated that there was direct interaction between miR-423-5p and CREBZF (Figure 7b). qRT-PCR detected the expression level of CREBZF mRNA, and Western blotting determined CREBZF protein level. It was apparent to see declining CREBZF mRNA level in 60 pairs of breast cancer patient tissues compared with that in normal tissues (Figure 7c). Meanwhile, reduced CREBZF protein level was detected in 8 pairs of normal tissues (Figure 7d). These findings indicated that miR-423-5p negatively targeted CREBZF and that down-regulated CREBZF level was associated with breast cancer development.

### 3.7. CREBZF knockdown could reverse the inhibitory effect mediated by miR-423-5p inhibitor on breast cancer cells

**Figure 8.**
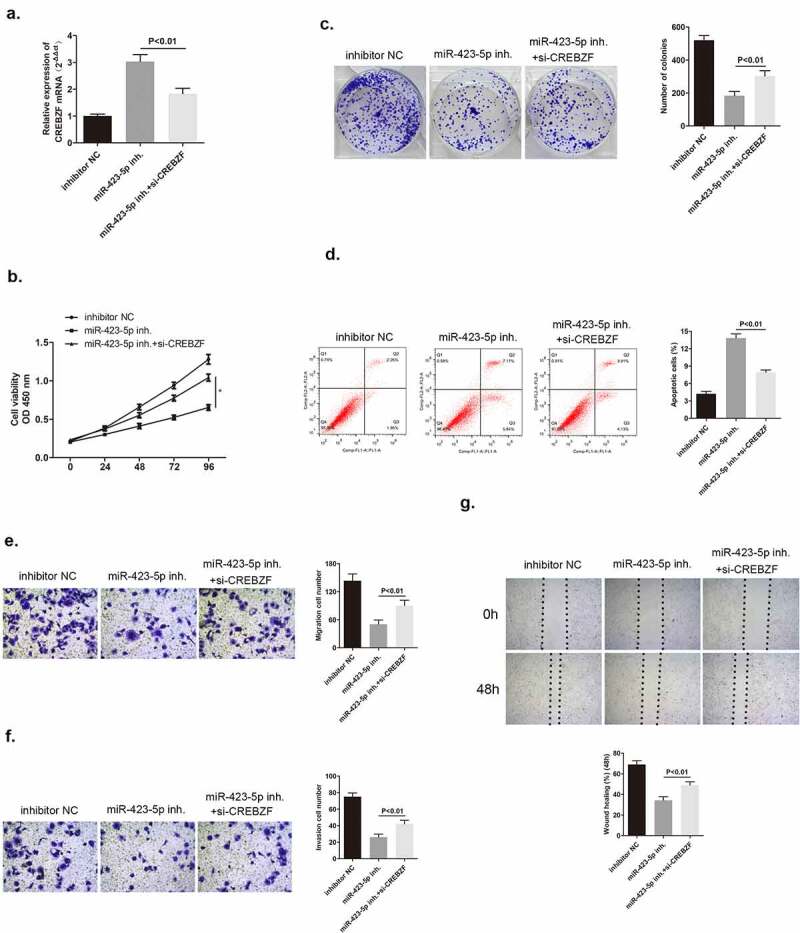
CREBZF knockdown reversed the effect of down-regulated miR-423-5p level on MCF-7 cells, and facilitated cancer cell development. (a) CREBZF mRNA level in the three groups after transfection as detected by qRT-PCR. (b) CCK-8 assay evaluated cell viability. (c) Colony formation assay identified the ability of clone formation. (d) Apoptosis ability accessed by flow cytometry. **(E&F)** The ability of migration and invasion was determined by transwell assay. (g) Wound healing assay was performed to describe migration capacity. Inhibitor NC, inhibitor negative control, with MCF-7 cells transfected with inhibitor NC plasmid; miR-422-5p inhi., miR-422-5p inhibitor, with MCF-7 cells transfected with miR-422-5p inhibito*r plasmid*; miR-422-5p inhi. + si-CREBZF, miR-422-5p inhibitor + siRNA of CREBZF, with MCF-7 cells transfected with miR-422-5p inhibito*r plasmid* and siRNA of CREBZF plasmid.

We have already confirmed that CREBZF was a target of miR-423-5p. There was a hypothesis that CREBZF may participate in miR-423-5p/MBNL1-AS1 axis and therefore, influence cell development. To explore its explicit effects on tumor cells, three groups of different plasmid transfection in MCF-7 cells were included. After administering si-CREBZF plasmid to miR-423-5p inhibitor transfection cells, CREBZF mRNA expression level was reduced compared with that in the miR-423-5p inhibitor group as observed via qRT-PCR (Figure 8a). Several experiments including CCK-8, colony formation assay, flow cytometry assay, transwell assay, and wound healing assay were conducted, and rate of MCF-7 cell viability was found to be suppressed in miR-423-5p inhibitor group, which was consistent with the former findings. Nevertheless, in si-CREBZF + miR-423-5p inhibitor group, which had lower CREBZF level, this suppressive effect was counteracted (Figure 8b,c). Similarly, cell apoptosis ability was weakened in si-CREBZF + miR-423-5p inhibitor group compared with that in the miR-423-5p inhibitor group (Figure 8d). The migration and invasion capacities of cancer cells were strengthened because of declining the CREBZF level in si-CREBZF + miR-423-5p inhibitor group compared with that in the miR-423-5p inhibitor group (Figure 8e-g). In conclusion, CREBZF knockdown could reverse the suppressive effect on MCF-7 cell proliferation, migration, and invasion brought about by miR-424-5p inhibitor and CREBZF could serve as a tumor suppressor.

### 3.8. MBNL1-AS1 influenced PI3K/AKT pathway by regulating CREBZF

**Figure 9.**
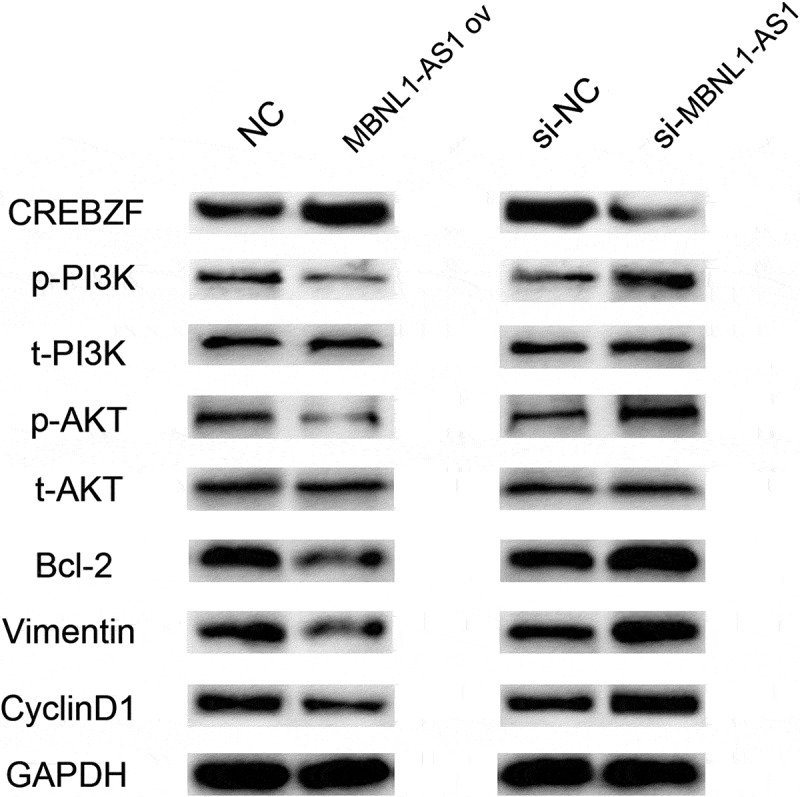
Western blotting results showed that MBNL1-AS1 affected PI3K/Akt pathway-related proteins through CREBZF. The phosphorylation of PI3K and Akt was changed, whereas the total PI3K and Akt remained the same, and further affected apoptosis pathway-related protein (Bcl-2), proliferation pathway-related protein (CyclinD1), and EMT pathway-related protein (Vimentin). NC, negative control, with MCF-7 cells transfected with blank plasmid; MBNL1-AS1 ov, MBNL1-AS1 overexpression, with MCF-7 cells transfected with MBNL1-AS1 overexpression plasmid; Si-NC, negative control of siRNA, with BT474 cells transfected with negative control of siRNA; si-MBNL1-AS1, siRNA of MBNL1-AS1, with BT474 cells transfected with siRNA of MBNL1-AS1.

To date, several studies have investigated PI3K/AKT, as a proto-oncogene, which has long been a question of great interest in a wide range of fields because it regulates various cell functions including metabolism, growth, proliferation, survival, transcription, and protein synthesis [18,19]. We applied Western blotting to determine the associated proteins levels to evaluate whether MBNL1-AS1/miR-423-5p/CREBZF axis influenced the PI3K/AKT pathway and was associated with the downstream proteins. Figure 9 shows that MBNL1-AS1 overexpression led to higher CREBZF level and down-regulated phosphorylated PI3K/AKT proteins (p-PI3K, p-AKT), whereas no change was observed in the total proteins (t-PI3K, t-AKT). Furthermore, the associated downstream proteins including apoptosis-associated protein Bcl-2, proliferation-associated protein CyclinD1, and EMT-associated protein Vimentin were examined. Lower Bcl-2, CyclinD1, and Vimentin levels were observed in MBNL1-AS1 ov group. In contrast, when MBNL1-AS1 was knocked down, CREBZF level was reduced, p-PI3K and p-AKT levels were increased, and the total PI3K and AKT levels remained the same. Bcl-2, CyclinD1, and Vimentin were up-regulated compared with the control group. Based on the abovementioned results, it can be concluded that MBNL1-AS1 influenced PI3K/AKT pathway by regulating CREBZF.

## 4. Discussion

Breast cancer is the most common malignancy among women, which is highly heterogeneous in nature. LncRNA dysfunction is closely associated with the pathological process of various diseases [20], and plays an important role in the occurrence, development and diagnosis, and treatment of breast cancer. Previous studies have reported the importance of abnormally up-regulated lncRNA expressions in breast cancer. For instance, BCAR4-induced tamoxifen resistance depends on the presence of ERBB2 (HER-2) and ERBB3 receptors [21]; H19 could enhance breast cancer cell proliferation and migration by activating Akt and Erk [22]; HOTAIR could interact with PRC2 to reprogram chromatin states in epigenetic gene silencing to promote cancer migration [23]; further, CASC7 suppressed malignant behaviors of breast cancer by regulating miR-21-5p/FASLG axis [13]. Our study revealed that low MBNL1-AS1 expression was found to indicate breast cancer development, decreasing MBNL1-AS1 level was detected both in breast cancer tissues and cell lines. Meanwhile, both *in vitro* gain-of-function and loss-of-function experiments and *in vivo* animal experiments collectively confirmed that MBNL1-AS1 functioned as a tumor suppressor by inhibiting cancer cell proliferation and migration and accelerating cell apoptosis.

In addition to directly participating in the regulation of gene expression, lncRNA can also affect the abundance of target gene mRNA by adsorbing miRNA, and thus affecting its protein level [11]. Studies have shown that many lncRNAs can sponge miRNAs because the structure of most lncRNAs has similarities with mRNAs; accordingly, the mode of regulating gene expression is more diverse and extensive, and will not be interfered by translation [24]. Peng et al. proved that lncRNA MEG3 inhibited the proliferation, migration, and invasion of gastric cancer cells by competitively binding to the miR-181 family [25]. In colon cancer, lncRNA H19 competitively combined with miR-138 and miR-200a, up-regulating the expression of key genes in EMT (VIM, ZEB1, and ZEB2), and promoting the progress of EMT [26]. The results of this study exhibited that MBNL1-AS1 directly sponged miR-423-5p through bioinformatics analysis and dual luciferase reporter assay. It was reported that miR-423-5p can promote autophagy in cancer cells and is increased in the serum of hepatocarcinoma patients treated with sorafenib [27]; miR-423-5p also regulated cell proliferation and invasion by targeting trefoil factor 1 in gastric cancer cells [28]; miR-423 further played a role in promoting breast cancer invasion by activating NF-κB signaling [29].

The current study discovered that miR-423-5p could counteract the inhibitory effect on cancer cell promotion brought about by MBNL1-AS1, indicating that miR-423-5p played a positive role in cancer promotion. Further, CREBZF, a member of the mammalian ATF/CREB family of transcription factors, was found to be a target of miR-423-5p. CREBZF knockdown could impair the deterioration of cancer cell growth mediated by low miR-423-5p expression.

In addition, dysregulation of the PI3K/AKT signaling pathway was observed in a variety of human diseases, including cancer, diabetes, cardiovascular diseases, and neurological diseases [30]. We performed Western blotting found that MBNL1-AS1 influenced PI3K/AKT pathway, which was relevant to cell proliferation and apoptosis, by regulating CREBZF. The abovementioned evidence supported the crucial role of abnormally low levels of MBNL1-AS1 in breast cancer. Also, the associated regulation network partly explained that MBNL1-AS1 exerted its function by regulating miR-423-5p/CREBZF axis, which can be a novel biomarker of breast cancer.

## 5. Conclusion

In summary, our work illustrated the tumor suppressor role of MBNL1-AS1 in breast cancer via the upregulation of miR-423-5p-targeted CREBZF. Thereby, the evidence points that a complete understanding of MBNL1-AS1/miR-423-5p/CREBZF axis in regulating breast cancer development is required, and that it could be seen as a biomarker in predicating survival of breast cancer patients. CREBZF was negatively regulated by miR-423-5p. Through several *in vitro* experiments, CREBZF knockdown was found to impair the deterioration of cancer cell growth mediated by low miR-423-5p expression. Also, MBNL1-AS1 was found to influence PI3K/AKT pathway, which was relevant to cell proliferation and apoptosis, by regulating CREBZF.

## Data Availability

The data of this study could be requested from the corresponding authors (nqcuser@163.com or kjljy@163.com).

## References

[1] Siegel RL, Miller KD, Jemal A. Cancer statistics, 2020. CA Cancer J Clin. 2020;70(1):7–30. 31912902 10.3322/caac.21590

[2] Okazaki Y, Furuno M, Kasukawa T, et al. Analysis of the mouse transcriptome based on functional annotation of 60,770 full-length cDNAs. Nature. 2002;420(6915):563–573. 12466851 10.1038/nature01266

[3] Batista PJ, Chang HY. Long noncoding RNAs: cellular address codes in development and disease. Cell. 2013;152(6):1298–1307. 23498938 10.1016/j.cell.2013.02.012 PMC3651923

[4] Zhu M, Wang Q, Tian P, et al. A long non-coding RNA specifically expressed in early embryos programs the metabolic balance in adult mice. Biochim Biophys Acta (BBA) - Mol Basis Dis. 2021;1867(1):165988. 10.1016/j.bbadis.2020.165988 33059001

[5] Wapinski O, Chang HY. Long noncoding RNAs and human disease. Trends Cell Biol. 2011;21(6):354–361. 21550244 10.1016/j.tcb.2011.04.001

[6] Choi M, Lu YW, Zhao J, et al. Transcriptional control of a novel long noncoding RNA Mymsl in smooth muscle cells by a single Cis-element and its initial functional characterization in vessels. J Mol Cell Cardiol. 2020;138:147–157. 31751568 10.1016/j.yjmcc.2019.11.148 PMC7036038

[7] Tang R, Qi Q, Wu R, et al. The polymorphic terminal-loop of pre-miR-1307 binding with MBNL1 contributes to colorectal carcinogenesis via interference with Dicer1 recruitment. Carcinogenesis. 2015;36(8):867–875. 25977444 10.1093/carcin/bgv066

[8] Zhu K, Wang Y, Liu L, et al. Long non-coding RNA MBNL1-AS1 regulates proliferation, migration, and invasion of cancer stem cells in colon cancer by interacting with MYL9 via sponging microRNA-412-3p. Clin Res Hepatol Gastroenterol. 2020;44(1):101–114. 31255531 10.1016/j.clinre.2019.05.001

[9] Wei X, Wang B, Wang Q, et al. MiR-362-5p, which is regulated by long non-coding RNA MBNL1-AS1, promotes the cell proliferation and tumor growth of bladder cancer by targeting QKI. Front Pharmacol. 2020;11(164). DOI:10.3389/fphar.2020.00164 PMC7063466 32194406

[10] Ye W, Lv Q, Wong CK, et al. The effect of central loops in miRNA:MRE duplexes on the efficiency of miRNA-mediated gene regulation. PLoS One. 2008;3(3):e1719. 18320040 10.1371/journal.pone.0001719 PMC2248708

[11] Salmena L, Poliseno L, Tay Y, et al. A ceRNA hypothesis: the rosetta stone of a hidden RNA language? Cell. 2011;146(3):353–358. 21802130 10.1016/j.cell.2011.07.014 PMC3235919

[12] Lopez-Mateo I, Villaronga MA, Llanos S, et al. The transcription factor CREBZF is a novel positive regulator of p53. Cell Cycle. 2012;11(20):3887–3895. 22983008 10.4161/cc.22133 PMC3495830

[13] Wang G, Duan P, Liu F, et al. Long non-coding RNA CASC7 suppresses malignant behaviors of breast cancer by regulating miR-21-5p/FASLG axis. Bioengineered. 2021;12(2):11555–11566. 34889164 10.1080/21655979.2021.2010372 PMC8809951

[14] Wang G, Bai X, Jiang G, et al. GIT1 overexpression promotes epithelial-mesenchymal transition and predicts poor prognosis in hepatocellular carcinoma. Bioengineered. 2021;12(1):30–43. 33258389 10.1080/21655979.2020.1855914 PMC8806235

[15] Tang F, Lu Z, Wang J, et al. Competitive endogenous RNA (ceRNA) regulation network of lncRNAs, miRNAs, and mRNAs in Wilms tumour. BMC Med Genomics. 2019;12(1):194. 31842887 10.1186/s12920-019-0644-y PMC6915924

[16] Hai T, Hartman MG. The molecular biology and nomenclature of the activating transcription factor/cAMP responsive element binding family of transcription factors: activating transcription factor proteins and homeostasis. Gene. 2001;273(1):1–11. 11483355 10.1016/s0378-1119(01)00551-0

[17] Xie YB, Lee OH, Nedumaran B, et al. SMILE, a new orphan nuclear receptor SHP-interacting protein, regulates SHP-repressed estrogen receptor transactivation. Biochem J. 2008;416(3):463–473. 18657049 10.1042/BJ20080782

[18] Bozulic L, Hemmings BA. PIKKing on PKB: regulation of PKB activity by phosphorylation. Curr Opin Cell Biol. 2009;21(2):256–261. 19303758 10.1016/j.ceb.2009.02.002

[19] Chen Y, Huang L, Dong Y, et al. Effect of AKT1 (p. E17K) hotspot mutation on malignant tumorigenesis and prognosis. Front Cell Dev Biol. 2020;8:573599. 33123537 10.3389/fcell.2020.573599 PMC7573235

[20] Maass PG, Luft FC, B?hring S. Long non-coding RNA in health and disease. J Mol Med. 2014;92(4):337–346. 24531795 10.1007/s00109-014-1131-8

[21] Xing Z, Lin A, Li C, et al. lncRNA directs cooperative epigenetic regulation downstream of chemokine signals. Cell. 2014;159(5):1110–1125. 25416949 10.1016/j.cell.2014.10.013 PMC4266991

[22] Vennin C, Spruyt N, Dahmani F, et al. H19 non coding RNA-derived miR-675 enhances tumorigenesis and metastasis of breast cancer cells by downregulating c-Cbl and Cbl-b. Oncotarget. 2015;6(30):29209–29223. 26353930 10.18632/oncotarget.4976 PMC4745721

[23] Gupta RA, Shah N, Wang KC. Long non-coding RNA HOTAIR reprograms chromatin state to promote cancer metastasis. Nature. 2010;464(7291):1071–1076. 20393566 10.1038/nature08975 PMC3049919

[24] Zeng Z, Huang H, Huang L, et al. Regulation network and expression profiles of Epstein-Barr virus-encoded microRNAs and their potential target host genes in nasopharyngeal carcinomas. Sci China Life Sci. 2014;57(3):315–326. 24532457 10.1007/s11427-013-4577-y

[25] Cordiali-Fei P, Trento E, Giovanetti M, et al. Analysis of the ORFK1 hypervariable regions reveal distinct HHV-8 clustering in Kaposi’s sarcoma and non-Kaposi’s cases. J Exp Clin Cancer Res. 2015;34(1):1. 25592960 10.1186/s13046-014-0119-0 PMC4311464

[26] Liang W-C, Fu W-M, Wong C-W, et al. The lncRNA H19 promotes epithelial to mesenchymal transition by functioning as miRNA sponges in colorectal cancer. Oncotarget. 2015;6(26):22513–22525. 26068968 10.18632/oncotarget.4154 PMC4673179

[27] Stiuso P, Potenza N, Lombardi A, et al. MicroRNA-423-5p promotes autophagy in cancer cells and is increased in serum from hepatocarcinoma patients treated with sorafenib. Mol Ther Nucleic Acids. 2015;4:e233. 25782064 10.1038/mtna.2015.8

[28] Liu J, Wang X, Yang X, et al. miRNA423-5p regulates cell proliferation and invasion by targeting trefoil factor 1 in gastric cancer cells. Cancer Lett. 2014;347(1):98–104. 24486742 10.1016/j.canlet.2014.01.024

[29] Dai T, Zhao X, Li Y, et al. miR-423 promotes breast cancer invasion by activating NF-κB signaling. Onco Targets Ther. 2020;13:5467. 32606763 10.2147/OTT.S236514 PMC7297514

[30] Carnero A, Blanco-Aparicio C, Renner O, et al. The PTEN/PI3K/AKT signalling pathway in cancer, therapeutic implications. Curr Cancer Drug Targets. 2008;8(3):187–198. 18473732 10.2174/156800908784293659

